# Sources of individual variation in plasma testosterone levels

**DOI:** 10.1098/rstb.2007.0001

**Published:** 2007-11-28

**Authors:** Bart Kempenaers, Anne Peters, Katharina Foerster

**Affiliations:** 1 Department of Behavioural Ecology and Evolutionary Genetics, Max Planck Institute for OrnithologyPO Box 1564, 82305 Seewiesen, Germany; 2 Research Group Evolution of Sexual Signals, Max Planck Institute for OrnithologyVogelwarte Radolfzell, 78315 Radolfzell am Bodensee, Germany

**Keywords:** hormone, heritability, behavioural syndrome, plasticity, genetic effects, maternal effects

## Abstract

The steroid hormone testosterone (T) plays a central role in the regulation of breeding in males, because many physiological, morphological and behavioural traits related to reproduction are T dependent. Moreover, in many seasonally breeding vertebrates, male plasma T levels typically show a pronounced peak during the breeding season. While such population-level patterns are fairly well worked out, the sources and the implications of the large variability in individual T levels within the seasonal cycle remain surprisingly little understood. Understanding the potential sources of individual variation in T levels is important for behavioural and evolutionary ecologists, for at least two reasons. First, in ‘honest signalling’ theory, T is hypothesized to play a critical role as the assumed factor that enforces honesty of the expression of sexually selected quality indicators. Second, T is often considered a key mediator of central life-history trade-offs, such as investment in survival versus reproduction or in mating versus parental care. Here, we discuss the patterns of within- and between-individual variation in male plasma T levels in free-living populations of birds. We argue that it is unclear whether this variability mainly reflects differences in underlying individual quality (intrinsic factors such as genetic or maternal effects) or in the environment (extrinsic factors including time of day, individual territorial status and past experience). Research in avian behavioural endocrinology has mainly focused on the effects of extrinsic factors, while other sources of variance are often ignored. We suggest that studies that use an integrative approach and investigate the relative importance of all potential sources of variation are essential for the interpretation of data on individual plasma T levels.

## 1. Introduction

### (a) Testosterone and behavioural ecology

Steroid hormones such as testosterone (T) influence or regulate the development or expression of suites of physiological, morphological and behavioural traits that are closely related to male fitness (reviewed in Adkins-Regan 2005). For example, in birds, it has been reported that plasma T levels correlate with testes size (e.g. Denk & Kempenaers 2005; Garamszegi *et al*. 2005), ornament size or colour (e.g. Buchanan *et al*. 2001; Redpath *et al*. 2006), territorial and aggressive behaviour, including mate guarding (reviewed in Soma 2006), singing behaviour (e.g. Ketterson *et al*. 1992; Hunt *et al*. 1997; De Ridder *et al*. 2000; Foerster *et al*. 2002), courtship behaviour (e.g. Pröve 1978; Fusani *et al*. 2007) and, ultimately, mating success (Raouf *et al*. 1997; Redpath *et al*. 2006).

Evolutionary changes in the expression of these fitness-related traits might thus be mediated by indirect selection on the underlying hormonal mechanisms (Adkins-Regan 2008). To understand this, assume that T influences the development of a morphological trait under selection. Further assume that individual T regimes (such as circulating T levels, their temporal pattern or the density or affinity of T receptors) show heritable genetic variation. Selection on the morphological trait will then lead to indirect selection, favouring males with T regimes that optimize the development of the trait. Consequently, selection on the morphological trait might cause a correlated response in other T-dependent traits (e.g. aggressiveness), without direct selection on them (for a more detailed treatment of this issue, see McGlothlin & Ketterson (2008)).

Attempts to integrate the study of proximate mechanisms with that of ultimate functions of behaviour have led to a prominent role of T in at least two areas of behavioural ecology.

First, the T dependence of male sexually selected traits is central to the idea of honest signalling (Folstad & Karter 1992; Andersson 1994; Zahavi & Zahavi 1997). T is considered the honesty-enforcing factor, because the maintenance of high T levels may come at a cost of increased basal metabolic rate (Buchanan *et al*. 2001), increased levels of stress hormones (Ketterson & Nolan 1999), reduced immunocompetence (reviewed in Owen-Ashley *et al*. 2004; Roberts *et al*. 2004), decreased resistance to oxidative stress (Alonso-Alvarez *et al*. 2007) and, ultimately, reduced survival (e.g. Redpath *et al*. 2006). The idea is that only the highest quality individuals can ‘sustain’ the highest T levels. Thus, individual variation in T levels may reflect *variation in quality*.

Second, T can be seen as a mediator of life-history trade-offs, such as investment in self-maintenance (survival) versus reproduction or in allocating resources to mating versus parental care (Ricklefs & Wikelski 2002; Hau 2007). For example, high plasma T levels usually coincide with periods of intense male–male competition in the form of territory defence, access to females, paternity protection or sperm competition, whereas low levels of T usually coincide with periods of paternal care (Wingfield *et al*. 1990; Pinxten *et al*. 2007). Thus, individual variation in the duration of elevated T levels may reflect *variation in life-history strategies*.

Two further issues are highly relevant for understanding the relationship between variation in T levels and phenotypic traits and are unfortunately often ignored by ecologists. They are highlighted elsewhere in this issue (Ball & Balthazart 2008) and we will only mention them briefly here. First, in the context of sexual selection theory, studies on the costs and benefits of maintaining high or low T levels usually assume a dose-dependent relationship between T and fitness-related traits. However, this relationship can have different forms and the evidence for dose dependency appears limited (reviewed in Hews & Moore 1997; Adkins-Regan 2005). In fact, variation in the duration of the period of elevated T might be more important to understand costs/benefits of T than variation in absolute T levels (see §2*b*). Second, as stressed by many endocrinologists, hormones are only part of the endocrine system and investigating them in isolation can lead to misleading conclusions (Dufty *et al*. 2002). Hormonal responses occur only if the appropriate receptors are available in or on the cell. Receptors can vary in number, affinity and specificity, and these properties might differ systematically among individuals (Adkins-Regan 2005; Ball & Balthazart 2008). Moreover, the number of receptors is not static, but can be up- and downregulated. Thus, the threshold at which T affects a trait can probably change within individuals, for example, through changes in receptor density in particular brain regions (Canoine *et al*. 2007). Finally, steroid hormones circulate mostly bound to proteins that increase their solubility in the blood. However, it is the unbound hormone that is biologically active. Thus, changes in plasma-binding protein levels will alter hormone availability without changing overall plasma hormone concentrations (Dufty *et al*. 2002; Ball & Balthazart 2008). In summary, the biological relevance of absolute plasma T levels may only be understood when considering variation in other aspects of individual T regimes.

### (b) Hormones and behaviour: intrinsic and extrinsic sources of variation

Depending on the focus of the researcher, there are two distinct approaches to individual variation in hormone-dependent behavioural traits. The first approach is based on the view that behavioural responses under hormonal control (including T) show a rather fixed pattern within individuals, and that they tell us something about an individual's quality, ‘type’ or strategy. This view is common among behavioural ecologists, and it relies on the assumption that those traits (and hence the underlying hormone levels) are mainly shaped by intrinsic factors and show heritable genetic variation. Intrinsic factors can be additive or non-additive genetic effects, maternal effects or other effects of the environment during early development. Selection on T-dependent traits has been emphasized (e.g. Folstad & Karter 1992; Raouf *et al*. 1997; Foerster & Kempenaers 2004, 2005; Møller *et al*. 2005; Alonso-Alvarez *et al*. 2007), even though surprisingly little is known about the heritability of T levels, and only few studies have investigated the relationship between individual T levels and fitness. If hormone regimes depend on intrinsic factors, and if they have correlated effects on a suite of behavioural traits, they may cause ‘behavioural syndromes’ or ‘personalities’ (Sih *et al*. 2004). Thus, this view emphasizes the role of testosterone in creating specialized phenotypes (e.g. Gil & Faure 2007; Sellers *et al*. 2007).

The second approach is the almost exact opposite. Here, it is emphasized that individuals often have to respond quickly to environmental changes, and that hormones such as T allow behavioural flexibility (Oliveira 2004). This view—adopted by many behavioural endocrinologists—focuses on extrinsic effects on hormone levels, and it implies that T levels can vary widely within individuals depending on the social context and the (recent) individual history. Indeed, there is plenty of evidence to suggest that not only do hormones influence behaviour, but that behaviour also influences hormone levels. Behavioural feedback on endocrine function has been well studied in the context of social interactions, related to male–male competition and female choice (e.g. Wingfield *et al*. 1987; Hirschenhauser *et al*. 2003; Oliveira 2004; Goymann *et al*. 2007).

The distinction between both approaches has rarely been made explicit, perhaps owing to a lack of communication between research fields. It is essentially different from the traditional distinction between organizational and activational effects of hormones (Arnold & Breedlove 1985; Moore *et al*. 1998). *Organizational effects* are those that occur during a critical or sensitive period in the early development of an individual and cause irreversible changes in the phenotype. *Activational effects* of hormones occur during adulthood and cause temporary changes in an individual's phenotype. Organizational effects are only relevant to the current discussion if they influence hormone regimes later in life (and then qualify as an intrinsic factor), whereas activational effects may depend on both intrinsic and extrinsic factors.

### (c) Defining the key questions and the scope of this paper

To better understand individual variation in hormone levels, we suggest combining the above two approaches in a more general framework. Individual hormone levels, the timing and duration of the period over which elevated hormone levels are maintained or the level of expression of a hormone-dependent behaviour are all quantitative traits. The observed variance of quantitative traits can be partitioned into genetic, maternal, environmental and residual (unexplained) variance. The variance due to genetic and maternal effects, as well as that due to environmental effects during early development (intrinsic factors), will contribute to perceived differences in individual quality or strategy. Environmental variance is induced by extrinsic factors such as the current social context, and thus contributes to flexible responses of individuals to environmental changes. Intrinsic and extrinsic factors are likely to interact in shaping the observed trait variance. To date, extrinsic effects on hormone regimes have received much more attention than intrinsic effects. Studies of intrinsic effects would benefit behavioural ecologists and endocrinologists alike, because they would (i) help interpret existing data, (ii) facilitate designing better experiments, and (iii) test the widely used assumption of intrinsic, evolvable effects on hormone regimes.

In §2 we first distinguish between two types of individual variation in plasma T levels. Then, in §3, we discuss the main factors that are presently known to influence variation in plasma T levels within and between individuals. We do this to call attention to the multitude of potentially confounding factors in studies on plasma T levels, and to those factors that are presently less intensively studied. In §4 we point to problems that may arise from underestimating or ignoring some of these factors. As an example, we discuss how data from simulated territorial intrusion experiments may be misinterpreted if intrinsic sources of individual variation in plasma T levels are ignored. Finally, in §5, we discuss the general implications for field studies and present suggestions and challenges for future research.

We focus on studies of variation in plasma T levels in populations of birds, simply because there is a rich literature available and birds feature prominently in the field of behavioural ecology. We also limit our discussion to variation in T levels in males. Although population-level patterns of circulating plasma T levels are fairly well studied (e.g. Garamszegi *et al*. 2005; Goymann *et al*. 2007), the sources and implications of the huge variability in individual T levels observed in natural populations remain surprisingly poorly understood (Adkins-Regan 2005).

Most of the issues we address here should be of more general relevance. First, there is a strong interest in the effects of T on females (e.g. McGlothlin *et al*. 2004; Zysling *et al*. 2006) and in understanding the evolutionary consequences of sex-specific effects (Møller *et al*. 2005; Ketterson 2007). Second, our discussion can easily be extended to other hormones. For example, several studies have focused on understanding variation in the endocrine response to stress (e.g. Müller *et al*. 2006), and selection lines have shown that part of this variation is heritable (Evans *et al*. 2006). Third, individual variation in other aspects of the endocrine system interacts with hormone-level variation to tune trait responses (see Ball & Balthazart 2008). Although currently less accessible to ecologists, individual variation in receptor density and affinity or in binding protein activity may have similar sources as those discussed here for variation in hormone levels.

## 2. Individual variation in plasma T concentrations

**Figure 1:**
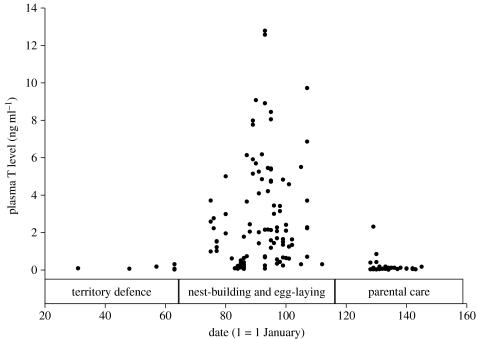
Plasma T levels in relation to capture date for blue tits (*Cyanistes caeruleus*) from a population breeding in nest-boxes in a deciduous forest (Kolbeterberg) in Vienna, Austria. Data are from the years 1998–2000 (Foerster *et al*. 2002) and 2002–2003 (Peters *et al*. 2006). The data shown (*N*=159) are from 132 individuals (one to four samples per individual), caught at different times of the day and using different methods (see text).

In seasonally breeding birds, plasma T in males typically shows a low baseline level during the non-breeding season (often including the period of paternal care) and one or more periods with elevated levels during the breeding season (reviewed in Goymann *et al*. 2007; see figure 1 for an example based on data from our blue tit, *Cyanistes caeruleus*, population).

**Figure 2:**
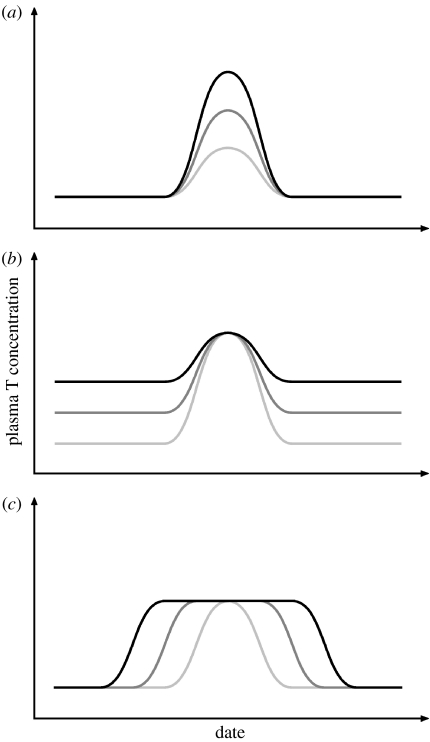
Theoretical seasonal changes in plasma T levels to illustrate three sources of variation. (*a*) Variation in T levels during the period of elevated T. (*b*) Variation in baseline T levels during the non-breeding season. (*c*) Variation in the duration of the period of elevated T. Note that, in reality, a combination of these types of variation is most likely and that in (*c*) variation in the timing of the increase in T may be independent of variation in the timing of the decline in T levels.

Here, we distinguish between two types of individual variation in T levels (figure 2). First, there is variation in the magnitude of individual T levels during a particular time period, either during breeding (variation in elevated T levels; figure 2*a*) or during non-breeding (variation in baseline T levels; figure 2*b*). Second, there is individual variation in the duration of the period in which elevated T levels are maintained (temporal variation; figure 2*c*), whereby this variation can be in the timing of the increase in T or in the timing of the decrease back to baseline levels, or in both. As a result, single samples from an individual can be difficult to interpret, whereas individual patterns over the breeding season are more meaningful, albeit much harder to obtain (cf. figures 1 and 2).

Distinguishing between variation in magnitude (at particular times) and in temporal expression is essential because these traits

- may differ in the relative importance of different sources of variance,
- may each underlie different specific selection pressures, and
- may show differences in potential to respond to selection (evolvability), depending on the magnitude of additive genetic variation for these traits.

Consequently, studies on T variation should address explicitly which of these two traits is being analysed. For example, many correlational studies analysed within-period variation in T levels, whereas many experiments used T implants to extend the duration of the period of elevated T, and this may explain some apparently contradictory results (see Kunc *et al*. 2006).

### (a) Variation in the magnitude of breeding and baseline plasma T levels

Average T levels during the breeding season differ substantially among species or populations, and this variation has been linked to variation in sexual selection (Garamszegi *et al*. 2005) or in overall life-history characteristics (Ricklefs & Wikelski 2002). However, the range of T values measured among individuals within a population often matches or even exceeds the range of mean T values between populations or species. There is remarkable variation in T levels during the period of elevated T, even when comparing individuals that do not obviously differ in status. For example, in our blue tit population in Vienna (Austria), there is a 200-fold difference between the individual with the highest T level and the one with the lowest T level during the period of elevated T (figure 1). A more extreme example is found in the pectoral sandpiper (*Calidris melanotos*): in a population of reproductively active males in Barrow (Alaska), the highest T level is more than 800 times higher than the lowest T level (Steiger *et al*. 2006; B. Kempenaers 2005, 2006, unpublished data).

During the non-breeding season, T levels are usually much lower. Individual variation in non-breeding T levels has received much less attention, probably owing to methodological problems (T levels may be below the detection limit of the assay and measurement error may be substantial relative to the true variation in T levels). However, that does not necessarily mean that T levels at this time are not important (e.g. Canoine *et al*. 2007) or that the variation in magnitude is not biologically relevant. For example, autumn T levels in male house sparrows (*Passer domesticus*) correlated positively with the change in the size of the black bib during the moult (Buchanan *et al*. 2001), which is presumably a sexually selected trait. Whether individuals that have relatively high baseline T levels also have relatively high T levels during the breeding period is remarkably unknown; the only study we are aware of is the one on wild-caught house sparrows, which reported a positive correlation between spring and autumn T levels (Buchanan *et al*. 2001).

### (b) Temporal variation in plasma T levels

Not only the magnitude but also the seasonal profile of elevated plasma T levels clearly differs among species and populations, for example, depending on the number of broods produced per season or on the mating system (Wingfield *et al*. 1987). However, the annual period of elevated T may also vary among individuals within a population, and this could reflect variation in life-history strategies. Males that keep elevated levels for a longer period may start breeding earlier, may breed more often during a single season or may be able to fertilize females for a longer period of time (Raouf *et al*. 1997). Such individual variation can be due to variation in the timing of the post-breeding decline in T, whereby some males return to baseline T levels earlier than others (parental care phase; figure 1). Additionally, males could vary in the timing of the onset of the spring increase in T. Such temporal variation has, for example, been reported in the superb fairy-wren, *Malurus cyaneus* (Peters *et al*. 2001), where males can moult into their nuptial plumage months before egg-laying starts. This has fitness consequences, because early moulting males later become successful extra-pair sires (Dunn & Cockburn 1999). Experimental and correlational data suggest that the highly variable timing of moult is causally linked to an increase in testosterone (Peters *et al*. 2000). Thus, there is considerable variation among males in the duration of the period during which they sustain high T levels. In this species, males that showed elevated T levels over a longer period of time did not have higher T levels during breeding (Peters *et al*. 2001). To date, no other study presented data on the correlation between the duration and the magnitude of elevated T.

Interestingly, individual variation in the seasonal profile of T can also depend on the environment. For example, double-brooded male white-crowned and song sparrows showed no change in T levels when renesting after a successful nesting attempt, but showed a strong increase in T when renesting after clutch or brood loss through predation (Wingfield & Moore 1987).

## 3. Sources of individual variation in plasma T levels

Few, if any, studies have rigorously tested both intrinsic and extrinsic effects on the variation in T levels described previously. Below, we specifically discuss five sources of individual variation that we consider pivotal for the interpretation of male plasma T levels measured in the field. We restrict this discussion mostly to variation in the magnitude of T levels during the breeding season, but similar factors are also likely to influence temporal variation in T levels.

### (a) Genetic effects (intrinsic)

**Figure 3:**
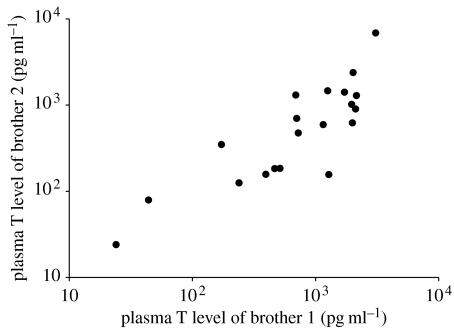
Plasma T levels of 20 pairs of brothers in a captive population of zebra finches (data from Pröve 1978). Note the logarithmic scale.

We are not aware of any study that has investigated the heritability of T levels in birds. Pröve (1978) reported plasma T levels for pairs of brothers in captive zebra finches (*Taeniopygia guttata*; figure 3). Based on these data, one can calculate a broad-sense heritability of *h*^2^=1.63 as the intraclass correlation coefficient divided by 0.5. Even a search for data from other taxa retrieves only a handful of studies. In humans, heritability estimates based on the degree of similarity between mono- and dizygotic twins (e.g. Meikle *et al*. 1997; Harris *et al*. 1998; Ring *et al*. 2005) or family members (Hong *et al*. 2001) were significant and varied between 0.16 and 0.69. Ring *et al*. (2005) found that additive genetic factors accounted for 57% of the variation in plasma T levels. This study is based on the largest sample and controls for confounding factors such as time of day and male age. In domestic pigs, the heritability of plasma T levels was estimated from father–son regressions (*h*^2^=0.37±0.16; Lubritz *et al*. 1991) and from a selection experiment: after 10 generations of artificial selection for divergent T levels, boars from the high-T line had levels that were approximately three times greater than those in individuals from the low-T line (Robison *et al*. 1994).

Information on heritability of T levels in natural populations is mostly lacking. The only study we are aware of reports full-sib comparisons in male garter snakes (*Thamnophis sirtalis*) at three ages (King *et al*. 2004). In the older snakes, heritabilities were highly significant and close to 1. Note that in this and all other studies mentioned above, the heritability values are probably inflated owing to common environmental (e.g. maternal) effects.

In conclusion, the available evidence is very limited, but clearly suggests that a substantial amount of the variation in plasma T levels may be explained by genetic differences among individuals. This also suggests that individual T levels should be, to some extent, repeatable across time and environments, which has rarely been tested (but see Jawor *et al*. 2006; Kralj-Fišer *et al*. 2007).

### (b) Maternal effects (intrinsic)

A variety of egg characteristics, including hormone levels, have been shown to affect offspring condition and growth. However, as a potential source of variance in individual T levels, such maternal effects are relevant only if they affect adult hormone levels, and this remains unclear.

Pioneering work by Schwabl (1993) showed that females transfer substantial and variable amounts of androgens to their eggs. Since then, many studies have investigated the effects of maternal androgens on early development of embryos and nestlings (Carere & Balthazart 2007). Recent evidence suggests that these effects may be long-lasting, so that they permanently shape the offspring phenotype, including traits that are thought to be mediated by adult T levels. For example, T-injected eggs in house sparrows resulted in adults with enlarged badge size (males only), which showed shorter latencies to approach and monopolize food (Strasser & Schwabl 2004). Similarly, in black-headed gulls (*Larus ridibundus*), juveniles from T eggs showed a higher frequency of aggressive and sexual displays and developed a more adult-like plumage, compared with those from control eggs (Eising *et al*. 2006). To what extent these long-lasting effects are induced by changes in the hypothalamus–pituitary–gonadal axis, the sensitivity to androgens and variation in neural structures remains to be shown (Groothuis *et al*. 2005; Groothuis & Schwabl 2008).

Avian maternal effects are not restricted to egg characteristics; they include all non-genetic effects on offspring trait variation, which are caused by differences between mothers. In species with biparental offspring care (such as many bird species), one should probably expand this to ‘parental effects’. Parents very much shape the early environment of their chicks and parental effects on fledgling traits are common. These effects might also have long-lasting consequences, including effects on adult hormone regimes. This could explain, for example, why experimentally induced variance in nestling food provisioning affected subsequent adult exploratory and aggressive behaviour (Carere *et al*. 2005). It seems feasible that neonatal conditions partly determine adult hormone levels, for example, through the effects on gonadal development. Understanding such parental effects seems crucial for the interpretation of individual variation in adult hormone levels.

### (c) Age effects (extrinsic)

Most studies on birds have reported that T levels did not vary with male age. Sometimes age was simply categorized as yearling versus older males (e.g. Belthoff *et al*. 1994; Beletsky *et al*. 1995; Schoech *et al*. 1996; Jawor *et al*. 2006; Peters *et al*. 2006; Madsen *et al*. 2007), but a number of studies on long-lived species confirmed that T did not vary across multiple age classes (Borgia & Wingfield 1991; Beletsky *et al*. 1995; Peters *et al*. 2002; Smith *et al*. 2005). However, a lack of age-related variation in T levels cannot be generalized: T levels in first-year males may decline earlier in the season compared with those in older males (Stunden *et al*. 1998) or yearlings may have lower peak T levels (e.g. Morton *et al*. 1990), possibly related to delayed breeding (Vleck & Brown 1999). Nevertheless, systematic studies to test whether and how plasma T levels change over the lifetime of individuals are rare. In Japanese quail, male T levels (and fertility) declined after a few years (Balthazart *et al*. 1984), suggesting that longitudinal analyses of the effect of age on T levels in free-living males will be worthwhile.

Not only may T levels vary with age, but age may also affect (i) seasonal patterns of variation in T levels and (ii) the relationship between T levels and fitness-related traits. In golden-collared manakins (*Manacus vitellinus*), T levels vary seasonally in adults (bright-plumaged), but not in juveniles (dull-plumaged), and T treatment during the breeding season increases display in juvenile but not in adult males (Day *et al*. 2007). Likewise, in blue tits, the relationship between individual plasma T levels and the intensity (chroma) of the UV/blue coloration of the crown feathers reversed with age, being positive in yearlings and negative in adults (Peters *et al*. 2006). The detection of such interactive patterns requires specific statistical testing and relatively large sample sizes.

### (d) Time-of-day effects (extrinsic)

Although some studies on birds have controlled for the time of capture as a confounding variable (e.g. Peters *et al*. 2001), systematic studies of diel variation in T appear rare. In male chicken, T levels were higher during the night compared with the daytime (Bachman *et al*. 1987), and a similar pattern was found in Lapland longspurs (*Calcarius lapponicus*), where T levels were elevated during the dim phases of the polar day (Hau *et al*. 2002). In contrast, T levels in the nocturnal Indian spotted owlet (*Athene brama*) showed the reversed pattern, that is, they were higher during the day (Guchhait & Haldar 1999), as has been observed in nocturnal mammals (Lerchl & Nieschlag 1995).

**Figure 4:**
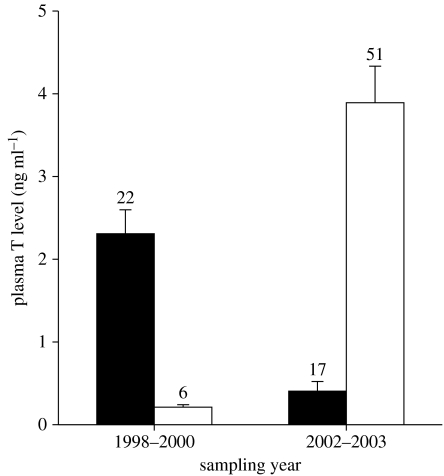
Plasma T levels of male blue tits from Kolbeterberg, Vienna (Austria), caught during the night (black bars; in the nest-box) or during the day (white bars; see text for details). During 1998–2000, T levels at night were significantly higher than those during the day (Mann–Whitney *U* test, *U*=4, *Z*=−3.47, *p*<0.0001); during 2002–2003, T levels at night were significantly lower than those during the day (*U*=11, *Z*=−5.98, *p*<0.0001). Sample sizes are indicated above the bars.

We compared plasma T levels of male blue tits that were caught at night or during the day and found significant differences (figure 4). Interestingly—but hard to explain—we also found that in the first study period (1998–2000), individuals that were caught at night had higher levels of T than individuals caught during the day, whereas this pattern was reversed in the second study period (2002–2003; figure 4). However, our study was not designed to test for circadian patterns of T, and a look at the details of the data collection highlights the potential importance of some other sources of T variation. For example, during 1998—2000, birds were caught during the day in food-baited Potter traps (Foerster *et al*. 2002), whereas, during 2002–2003, they were caught with mistnets after a simulated territorial intrusion (STI; with playback and a dummy; see Peters *et al*. 2006). One could argue that we measured higher levels in the second study because the STI caused an increase in T levels, but, in §4, we discuss that blue tits reacted to a prolonged STI with a decrease in T. We consider it possible that Potter traps attract a different blue tit phenotype than birds caught with an STI, and that individual-intrinsic factors (e.g. genetic or maternal effects) are responsible for the observed difference. However, T levels measured at night (between 20.00 and 02.00) also differed between the two study periods, for hitherto unknown reasons. In conclusion, previous work and our preliminary data suggest that it is worthwhile to study diel patterns of plasma T in birds. Apart from establishing whether such a pattern exists, one can also question its possible adaptive significance. For example, does daily variation in T levels (e.g. morning peak) relate to daily variation in song output (dawn chorus), territorial intrusions or copulation rate? Are daily fluctuations in T preparing individuals for periods of intense competition for fertilizations? These questions remain unanswered.

### (e) Effects of the social environment (extrinsic)

The idea that the behaviour of an individual and its interactions with other individuals feeds back on the individual's androgen levels has a long history and there is substantial empirical support for it in a variety of taxa (reviewed in Oliveira 2004). In male birds, T levels can be affected by individual mating opportunities, social status, aggressive encounters and other social interactions (including male–female interactions).

It is well established that individual male T levels increase when their mate becomes fertilizable (Wingfield *et al*. 1990), an effect that can be mimicked by oestradiol treatment of the female (Moore 1982; Runfeldt & Wingfield 1985). This effect is not limited to pairs: in polygynous red-winged blackbirds, T increases when any female on the male's territory becomes receptive (Johnsen 1998) and, in promiscuous superb fairy-wrens, male T levels covary with the number of fertile females in the population (Peters *et al*. 2001). The relationship between T and social status appears less predictable: subordinates in some group-living species have lower T during breeding, often in association with reduced reproductive ability (e.g. Schoech *et al*. 1996; Vleck & Brown 1999). However, T levels may be elevated when subordinates have mating opportunities, resulting in no or a small effect of male status (Reyer 1986; Peters *et al*. 2001, 2002). However, more elusive than seasonal variation in T levels associated with relatively stable social systems are short-term fluctuations in individual hormone levels due to recent events.

The idea that social experience feeds back onto hormone levels has been formalized in the ‘challenge hypothesis’ (Wingfield *et al*. 1987, 1990), which has been particularly influential in this field of research (more than 650 citations of the two key papers). The hypothesis has been modified several times (most recently by Goymann *et al*. (2007)), but the core idea has not changed. The hypothesis postulates the existence of different levels of circulating T: a constitutive, baseline or homeostatic level (*A*); a regulated, periodic, breeding level (*B*), which is induced by environmental cues such as an increase in day length and which is sufficient for the expression of spermatogenesis, secondary sexual traits and male reproductive behaviours; and a regulated, facultative, physiological maximum level (*C*), which can be induced by social interactions. Wingfield *et al*. (1990) further proposed that the hormonal response to social interactions can be estimated by the androgen responsiveness *R*, which equals (*C*−*A*)/(*B*−*A*). This allows a comparison of the responsiveness of individuals (or populations and species) to social challenges, because *R* is independent of variation in baseline or absolute maximum T levels. This framework has been used successfully to explain interspecific variation in hormonal responses in relation to the mating system and the number of broods per season, and to explain individual or population-level variation in hormone levels in relation to social stability, social or territorial status, breeding density and mating success (reviewed in Oliveira 2004; see also Goymann *et al*. 2007).

The main problem with the above approach is that, in practice, it might be very difficult to obtain reliable estimates of the average breeding level *B* and the maximum level *C* (level *A* can be measured during the non-breeding season). As an example, Goymann *et al*. (2007) provide estimates of the different levels of T for the blue tit (p. 466), based on data from Landys *et al*. (2007). They suggest that level *A* is non-detectable, that level *B* is the mean level measured during the parental care phase (0.6 ng ml^−1^) and that level C is the mean level that was measured during egg-laying (5.4 ng ml^−1^). However, inspection of figure 1 suggests that in most individuals, T levels during the parental care phase are similar to non-breeding baseline levels as observed in late winter or early spring, long before sexual activity starts. It can be argued that during the parental phase, levels of T are already declining from breeding levels (*B*) to baseline levels (*A*). Alternatively, one could point out that some reproductively active individuals showed equally low T levels during the nest-building and egg-laying periods (period of elevated T; figure 1), suggesting that we observed breeding levels (*B*) from late winter until early summer (across a four-month period; figure 1). Finally, it also seems rather arbitrary to take the mean T level measured during the early breeding (egg-laying) phase as an estimate of *C*. At least, it is unclear how this value relates to the maximum T level that can be induced by social interactions.

There is no easy way to solve these problems, but it is clear that an experimental approach might be helpful. Goymann *et al*. (2007) suggest a useful framework that may help to obtain more precise estimates of the effects of the social environment on T levels of individual males. Essentially, they suggest estimating different components of *R* separately. This could be achieved, for example, by measuring T levels in response to a simulated territorial intrusion (relative to control males), to exposure to a potential extra-pair female that either solicits or refuses to copulate (relative to exposure to the social mate) or to a standardized injection of GnRH (to estimate the physiological capacity of an individual to mount a T response; e.g. Robison *et al*. 1994; Jawor *et al*. 2006). However, the interpretation of data from these experiments will continue to pose problems if other sources of trait variance are disregarded. The acknowledgement and better understanding of individual variation in *B*, *C* and hence *R*, due to intrinsic and non-social extrinsic factors, will help in designing better experiments by ensuring a random distribution of these factors or, more effectively, by minimizing their variance.

In §4 we discuss one example of an experimental approach to measure an effect of the social environment: the response of individuals to simulated territorial intrusions. We discuss how the consideration of both intrinsic and extrinsic sources of variation in plasma T levels may affect the interpretation of the experimental data.

## 4. Interpretation of experimental data

**Figure 5:**
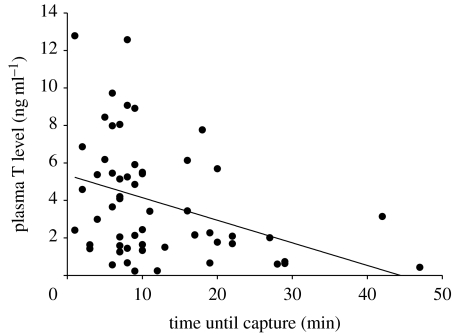
Plasma T levels in 57 male blue tits in relation to the time they were exposed to a simulated territorial intrusion (9 min playback, presentation of a dummy until capture). The negative relationship between plasma T and capture time is significant (Spearman's rank correlation *r*=−0.38, *p*=0.004). Data are from Peters *et al*. (2006). Blood sampling was completed within 3.5–12.5 min of capture.

The challenge hypothesis predicts that when birds are challenged by an intruder, or more generally, when they compete with other males, their T levels should increase. This has been tested experimentally by comparing T levels after simulating a territorial intrusion with those of control individuals that have not been challenged (e.g. Van Duyse *et al*. 2004), or by examining the change in T values in relation to the duration of a simulated intrusion (e.g. Wikelski *et al*. 1999). Studies that have used such an approach are reviewed in Landys *et al*. (2007). The results are mixed: some studies showed an increase in T levels (e.g. Wikelski *et al*. 1999), some showed no change (e.g. Meddle *et al*. 2002) and others showed a decline (e.g. Van Duyse *et al*. 2004; Landys *et al*. 2007). Landys *et al*. (2007) acknowledge the possible effects of differences in experimental design among studies, but they also suggested that there is a species-specific response depending on the number of broods: only multi-brooded species showed the predicted positive response. We argue that there are alternative explanations for the observed changes in T levels after simulated territorial intrusions. To illustrate this point, we show data from our own work on blue tits (figure 5).

We simulated a territorial intrusion by presenting a taxidermic mount of a male blue tit in the focal territory and using a 9 min playback of blue tit song (Peters *et al*. 2006). Mistnets had been put up around the dummy to catch the responding male. Figure 5 shows the time between the start of the simulated intrusion and capture, which ranged from 1 to 47 min. The challenge hypothesis predicts that T levels should increase, at least after the minimum time of 10 min required for a response (based on previous work, see Landys *et al*. (2007)). However, in our experiment, T levels declined with time until capture (figure 5). There are at least two fundamentally different explanations for this decline. (i) T levels of individuals declined over time. In practice, this would be difficult to show because one would need to catch the individual before and after the experiment. However, such an individual decline is expected based on the ‘winner–loser effect’ (Oliveira 2004). Aggressive encounters are common in natural situations, but are usually very brief with the territory owner winning the conflict. In the case of a prolonged simulated intrusion, however, the territory owner is unable to evict the intruder, and—at least in the case of a stuffed bird—the intruder will not even be impressed by the attacks (e.g. by trying to hide or show a submissive posture). Not surprisingly, this increases stress and hence the corticosterone levels in the territory owner (Landys *et al*. 2007), and perhaps also explains the decrease in T in this and other studies (e.g. Van Duyse *et al*. 2004). (ii) The observed effect arises because the time until capture is related to an individual's T level. This is not unlikely either, because individuals with high T might be more aggressive, more active or more dominant, and therefore they approach the dummy faster than individuals with lower T levels. Thus, the birds that are caught later might be inherently different from the birds that are caught earlier. This alternative can only be excluded if focal males are randomly assigned to experimental groups, which are exposed to intrusions of different duration. This is exactly what Landys *et al*. (2007) did: males were randomly assigned to either a 10 min or a 30 min intrusion treatment and T levels after treatment were compared with those in a control group of birds caught in baited walk-in traps. Landys *et al*. (2007) observed a decline in T levels with longer simulated intrusion, which is most likely due to individual changes in T. This example emphasizes the importance of carefully designed experiments.

Similar problems of interpretation arise in non-experimental tests of the challenge hypothesis. For example, Smith *et al*. (2005) studied plasma T levels in cliff swallow (*Petrochelidon pyrrhonota*) colonies of different sizes. They reported higher T levels in males from larger colonies, presumably because these males competed more for (extra-pair) matings than males from smaller colonies. However, the alternative hypothesis is that male T levels influenced settlement patterns (choice for colony size). Indeed, males with above-average T levels were more likely to choose a colony of the same size or larger in a later year, compared with males with below-average T levels (Brown *et al*. 2005).

## 5. Concluding remarks

In this paper, we argued that individual variation in plasma T levels, as measured in natural populations of birds, is often hard to interpret. This is partly due to the current dichotomy of scientific approaches to this topic, reflecting the two fundamentally different roles of T which have been suggested in the literature. On the one hand, variation in plasma T levels is seen as an intrinsic attribute of individuals, due to genetic, maternal or other early environmental effects, causing specialization into specific phenotypes. On the other hand, the focus lies on short-term variation in plasma T levels triggered by extrinsic factors, which might allow individuals to respond quickly and flexibly to environmental challenges. We suggested the adoption of a conceptual framework, where both intrinsic and extrinsic effects on individual variation in T levels are considered. To achieve this aim, future research could contribute in two ways.

First, we need to close the gaps in our knowledge on the impact of specific factors on individual hormone levels. Much research has been done on effects of the social environment. Age effects and time-of-day effects are routinely considered as covariates, but studies that specifically target these factors and estimate their importance for avian hormone regimes are scarce. Data from other taxa suggest that they are important and more information about their role in birds is needed.

The two intrinsic sources of individual variation, genetic and maternal (or more generally parental) effects, have mostly been neglected. We urgently need to gather information on the magnitude of additive genetic effects on individual T levels. Studies on the adaptive value of individual T-level variation need to be built on realistic, tested estimates of the heritability of T levels. Here, significant progress could be made through quantitative genetic studies, which have already contributed to the understanding of genetic trait variance in wild bird populations (Merilä & Sheldon 2001). Another fruitful approach might be to create selection lines for individuals with diverging T levels or with diverging timing of increase in T. The fact that this is possible is witnessed by a study on zebra finches, where individuals were selected for high or low stress (corticosterone) response (Evans *et al*. 2006).

Parental effects can be numerous and varied and we suggest approaching this problem from various angles. Experiments that already demonstrated parental effects on T-mediated traits in adults (see §3) could be repeated, to directly test effects on adult plasma T levels. New experiments could aim at manipulating conditions in early life which are mainly determined by parental qualities in the wild and which are also likely to influence adult hormone regimes. The most promising candidate factors for such experiments might be best determined through cooperative efforts of endocrinologists and behavioural ecologists. Finally, quantitative genetic studies on adult T levels would not only reveal the magnitude of heritable genetic variation, but also allow the estimation of the relative importance of direct parental effects.

As a second step towards an integrated conceptual framework on individual T-level variation, both intrinsic and extrinsic factors need to be considered when designing experiments or interpreting correlational data. Currently, studies on extrinsic factors rarely acknowledge the potential contribution of genetic variation or parental effects to variation in observed hormone levels. We pointed out how this can lead to the misinterpretation of extrinsic effects. Although it will be difficult to eliminate the effect of genetic variation in experiments that are designed to measure extrinsic effects, a first step would be the repeated sampling of individuals or sampling of individuals with known relatedness. On the other hand, experiments targeting intrinsic factors need to consider potential confounding environmental and social effects. Finally, we should bear in mind that different factors may not only have independent effects on individual T-level variation. Interactions between intrinsic and extrinsic effects are probable and will ultimately need to be addressed. For example, in humans, the relationship between individual T levels and cognition depends on social status (Newman *et al*. 2005). As mentioned in §3*c*, in birds, male age may affect relationships between T levels and phenotypic traits. Generally speaking, age effects may be independent from genetic effects (if they are correlated across different genotypes), but they may also interact with genetic effects (if different genotypes show different age-specific trait expressions). Although this problem seems too complex to tackle given the current knowledge of the sources of individual T-level variation, it stands as a reminder of the challenges we face in interpreting individual variation.

Even if we succeed in defining the relevant sources of T-level variation, the link between observed T levels and behavioural or other fitness-related traits is far from obvious. Although not the main focus of this paper, we briefly mentioned two further questions central to this problem (see §1; Ball & Balthazart 2008). (i) What is the form of the relationship between the level of plasma T and the development or maintenance of a trait? (ii) What is the biological relevance of absolute levels of plasma T in individuals?

Our final point is that there is a paucity of field studies that have assessed the long-term fitness effects of varying T levels. Such work could be particularly useful in populations that have served as model systems for studying other aspects of sexual selection. Reed *et al*. (2006) studied long-term fitness consequences of experimentally elevated T levels in dark-eyed juncos. The study is exceptional in several ways: it is based on data from nine breeding seasons and models effects on a variety of reproductive parameters (clutch size, number of nesting attempts, nest success and nestling growth), including loss and gain of extra-pair paternity and social mate choice, and effects on survival at every stage from fledgling to adult. One of the interesting outcomes is that the positive effect of high T on gaining extra-pair fertilizations should lead to selection for males with higher T. This might be countered by selection against these individuals through negative effects on females, but the study could not directly address this (Reed *et al*. 2006).

This paper is the result of a workshop that brought behavioural endocrinologists and behavioural ecologists together to discuss individual variation. We hope that—in the spirit of this event—our paper will stimulate research that advances both fields.
